# Sustaining a Regional Emerging Infectious Disease Research Network: A Trust-Based Approach

**DOI:** 10.3402/ehtj.v6i0.19957

**Published:** 2013-01-25

**Authors:** Pornpit Silkavute, Dinh Xuan Tung, Pongpisut Jongudomsuk

**Affiliations:** 1 Health Systems Research Institute, Ministry of Public Health, Nonthaburi, Thailand; 2 National Institute of Animal Sciences, Ministry of Agriculture and Rural Development Hanoi, Vietnam

**Keywords:** APEIR, pandemic preparedness, multi-country, multi-sectoral, multi-disciplinary, trust-based research network, emerging infectious disease, policy

## Abstract

The Asia Partnership on Emerging Infectious Diseases Research (APEIR) was initiated in 2006 to promote regional collaboration in avian influenza research. In 2009, the partnership expanded its scope to include all emerging infectious diseases. APEIR partners include public health and animal researchers, officials and practitioners from Cambodia, China, Lao PDR, Indonesia, Thailand and Vietnam. APEIR has accomplished several major achievements in three key areas of activity: (i) knowledge generation (i.e., through research); (ii) research capacity building (e.g., by developing high-quality research proposals, by planning and conducting joint research projects, by adopting a broader Ecohealth/OneHealth approach); and (iii) policy advocacy (e.g., by disseminating research results to policy makers). This paper describes these achievements, with a focus on the partnership's five major areas of emerging infectious disease research: wild migratory birds, backyard poultry systems, socio-economic impact, policy analysis, and control measures. We highlight two case studies illustrating how the partnership's research results are being used to inform policy. We also highlight lessons learned after five years of working hard to build our partnership and the value added by a multi-country, multi-sectoral, multi-disciplinary research partnership like APEIR.

## Introduction

In 2004–2005, outbreaks of highly pathogenic avian influenza (HPAI) in poultry were reported in eight countries in Southeast and East Asia: China, Cambodia, Thailand, Viet Nam, Indonesia, South Korea, Japan, and Lao PDR. The outbreaks caused serious damages to the poultry sector and to the regional economy (1). Institutions in the most severely affected Asian countries joined together to form the Asia Partnership on Avian Influenza Research (APAIR) as a means to improve the regional response to the threat of pandemic influenza. Upon the emergence of H1N1 (“swine flu”) in 2009, the network expanded its scope to include other emerging infectious diseases and was renamed the Asia Partnership on Emerging Infectious Diseases Research (APEIR). APEIR is also known as the Asia Emerging Infectious Disease (EID) research network (www.apeiresearch.net). Initially supported by Canada's International Development Research Centre (IDRC), the focus of the partnership has been on joint research activities and the translation of research results into policy and practice.

APEIR is a multi-country, multi-disciplinary, multi-sectoral research network whose focus is on regional priorities. The network is led and implemented by researchers and government officials from the region and includes representatives from more than 30 partner institutions (research institutes, universities, ministry departments). While primarily a research partnership, the network also advocates for policy and practice change in both animal and public health. Indeed, the two priorities are inter-linked. Policy advocacy provides a means to disseminate research findings, and scientific evidence from research studies assists in policy development.

The partnership has recently reviewed its functions and defined a new vision and mission. The APEIR vision is to be the leading knowledge and research network in Asia for emerging infectious diseases. Its mission is to develop a strong regional partnership in Asia that generates multi-disciplinary collaborative research on emerging infectious diseases and that facilitates communication and knowledge sharing among countries to reduce the threat of EIDs and the burden on these countries, especially on poor and marginalized groups in the region. APEIR's research mission is based on the Ecohealth paradigm, which is an ecosystem-based human health research approach that considers socio-economic, cultural, and environmental factors and is based on a set of six core principles (systems thinking, transdisciplinarity, multi-stakeholder participation, equity, sustainability, and knowledge for action) (2).

This article chronicles the history of APEIR and describes its governance and trust-based approach; major research activities and key achievements; and challenges for future sustainability.

## History, Governance, and APEIR's Trust-Based Approach

A unique feature of APEIR is its trust-based, bottom-up approach. The network research teams and topics were formed through a collaborative process, starting with each country holding its own multi-partner consultation meeting to identify national research priorities and mechanisms for partnership at national and regional levels. Then, national stakeholders from member countries convened to discuss the possibilities. At the stakeholder meeting, country teams presented their research ideas, including objectives, team composition (lead person and institutions), concrete ideas for implementation, means of information sharing and networking, and policy relevance. Topics were identified as regional priorities if they were selected and supported by at least three countries. All of this occurred during the first year of the partnership, in 2006. Also during its first year of existence and also reflecting APEIR's trust-based, bottom-up approach, partners began a joint discussion on governance of the partnership, including both its structure and mechanism (see below). Today, the trust-based approach is exemplified by the transparent communications among the partners and the fact that all partners’ contributions are considered and valued equally. Decisions are made after input from all partners.

During its second year of existence, in 2007, research proposals were finalized and funded and research projects initiated. Also during its second year, the partnership set up a regional Coordinating Office (CO), one of three key governance entities. Based at the Health Systems Research Institute (HSRI) in Nonthaburi, Thailand, the regional CO serves as the main communication hub among partners and coordinates and monitors the work of the research teams.

For the next few years (2008–2010), the research network was strengthened at both national and regional levels as collaborative research projects proceeded. Also during that period, APEIR developed a Strategic Plan 2010–2013 and identified three major, inter-related strategic directions: i) Knowledge generation and management: Support and share collaborative multi-disciplinary research on EIDs that transforms “tacit” knowledge into “explicit” knowledge through policy briefs and other products. ii) Capacity building: Strengthen the capacity of multi-disciplinary researchers, institutions and trust-based networks – within and among member countries. iii) Social and policy advocacy: Use strong collective social capital to advocate for appropriate social and policy responses, based on empirical evidence from research and practice.

### Governance

In addition to the CO, the other two key entities of APEIR's governance structure are the Partnership Steering Committee (SC) and the national research teams themselves. Again reflecting its trust-based, bottom-up approach, the SC was formed by asking each country group to identify institutional representatives to sit on the SC. The country groups were asked to identify policy-makers, not scientists, and from different sectors (i.e., one representative from public health, the other from an agricultural sector). Thus, the SC is comprised of 13 members: two representatives from each country, plus a chairperson recommended by the other SC members. The chair serves a two-year term. The SC provides overall guidance, coordination and supervision of the work of the partnership. It appoints and guides the activities of the CO; and creates a supportive environment for the emergence of research projects and other network activities. Some SC members serve on high-level national committees or expert panels on EIDs, enabling them to share relevant APEIR research findings and thereby inform and influence policy.

The three governance entities – the CO, the SC, and the national research teams – regularly interact, mainly though emails. Additionally, face-to-face meetings and workshops have also been organized. For example, workshops have been held for the research teams to report their project progress and findings to the SC and for comments and recommendations from the SC to be incorporated into research updates and final reports. SC meetings are held twice a year, during which the CO reports on partnership activity progress; and SC teleconferences are conducted as needed.

## Activities and Accomplishments

APEIR conducts a wide range of activities which, together, span its three strategic directions (i.e., knowledge generation and management, capacity building, and social and policy advocacy).

### Knowledge Generation and Management

Under SC supervision, the national research teams have collaborated on the design, implementation, and completion of five major IDRC-funded regional research projects (3):

1. In order to better understand the role of wild birds in spreading disease, APEIR formed a regional network for surveillance and monitoring of avian influenza viruses in migratory birds. The researchers concluded that major wild bird migration routes along the central Asia flyway overlap with areas that have experienced avian influenza outbreaks in poultry in Tibet, but that it is not clear whether the wild birds were the source of poultry infection (Text Box 1).
2. A multi-country APEIR team conducted an analysis of the socio-economic impact of human pandemic avian influenza outbreaks and control measures on small-scale and backyard poultry producers in Asia. The project revealed that the backyard poultry sector is resilient to shock even when the impact on livelihoods is considerable; and that the sector is likely to persist even if government policies call for a “restructuring” of the industry (See Text Box 2). By contrast, the small-scale commercial sector (i.e., smallholders whose livelihoods depend upon raising and selling poultry) is much more vulnerable to shock and needs government support to prevent bankruptcy and to assist restocking. However, farmers considered the compensation rate for culling of poultry during the HPAI outbreak to be far too small; the rate should be increased to discourage farmers from hiding or selling their infected poultry and to encourage farmers to apply control measures.
3. APEIR researchers conducted a study in five Asian countries on the characteristics and dynamics of backyard poultry systems in relation to reducing and managing avian influenza risks. The project found that biosecurity is generally quite low in both small-holder (100 percent) and larger commercial farms (70 percent).
4. An APEIR analysis of pandemic influenza preparedness policy identified variation in policy among countries and identified factors that influence policy formulation. The study concluded that scientific evidence does play a role in related discussions, but that national economic interest is also important (Text Box 2) (9).
5. Multi-country joint studies on the effectiveness of avian influenza control measures showed that: control of highly pathogenic avian influenza was achieved despite many control measures being implemented imperfectly; while vaccination in Vietnam and China was not expected to prevent (and did not prevent) all cases of infection, it almost certainly played a role in reducing both disease levels and the quantities of virus shed by vaccinated infected poultry; and, while poultry vaccination appears to have reduced the occurrence of outbreaks of poultry disease in Vietnam and China, it may be masking virus presence. Regarding the last finding, even where mandated by law, vaccination coverage is imperfect. Thus, the virus erupts from time to time. Reliance on mass vaccination may be leading to neglect of other measures.

These various research projects have generated a number of outputs, including books published in national languages, peer-reviewed scientific articles, reports, and presentations and conference papers. See the APEIR website for a list of published and on-line documents (10).

***Text Box 1*. Surveillance and monitoring of avian influenza in wild birds**

**Fig. 1:**
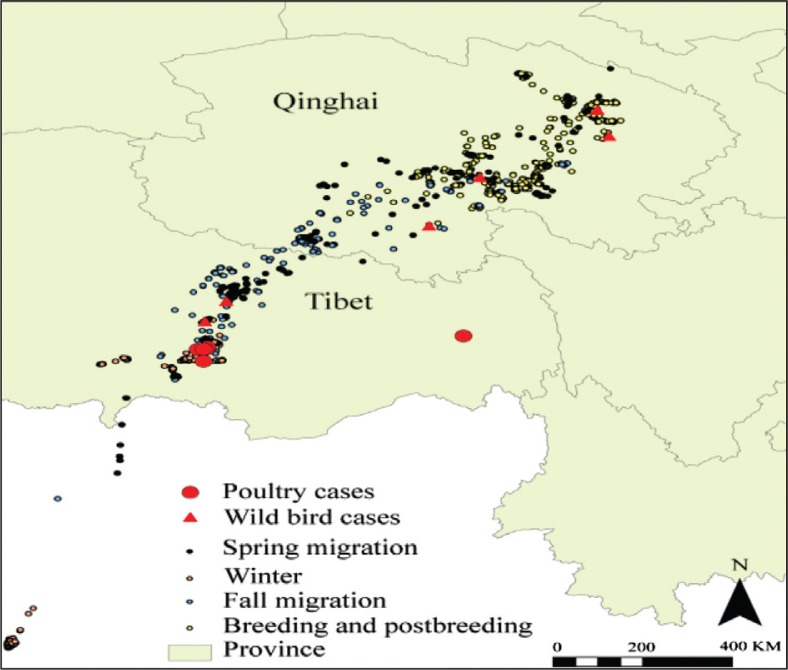
Locations of HPAI outbreak sites and wild bird movements along the Qinghai-Tibetan Plateau. Source: APEIR.

Among APEIR's first research activities was formation of a regional network for the surveillance and monitoring of avian influenza in migratory birds to help with the assessment, prevention, and control of cross-species influenza disease transmission (Figure 1) (4). A multi-country research team comprised of Cambodian, Thai, Indonesian and Chinese scientists consolidated findings about the role of wild birds in the transmission of HPAI and collected additional samples from wild birds. For example, findings from several countries demonstrate spatial links between outbreaks of HPAI in poultry and outbreaks in wild birds (e.g., 5–6). However, some findings also reveal weak temporal links between poultry and wild bird outbreaks; evidence from Thailand suggests that spread of the virus appears to be predominantly through poultry (not wild birds). Testing of healthy wild birds resulted in a low proportion of positive samples in all countries, again demonstrating that carriage of H5N1 HPAI virus by these birds probably occurs infrequently. Together, the findings confirm the need to segregate poultry from wild birds, but also demonstrate that, even in places where wild birds and poultry are co-located, wild birds may not necessarily be the source of infection in poultry (and vice versa) (7).

***Text Box 2*. From policy analysis to policy impact assessment**

Another of APEIR's first research activities was an analysis of national pandemic preparedness policies and plans among Asian countries. Funded by IDRC in 2007, APEIR analyzed policies regarding poultry vaccination and antiviral drugs in Thailand, Indonesia, and Vietnam. The research team found that the three countries’ policies shared some similarities but also had some differences; and that scientific evidence played a role in policy development, but so too did national economic interest, with the same scientific evidence being interpreted differently in different countries and different national approaches sometimes impeding regional efforts (4, 8).

More recently, APEIR started another IDRC-funded study in 2011 that aims to measure the impact of poultry production policies that have been implemented in several Asian countries as protection against avian influenza threats (8). Specifically, China, Indonesia, Thailand, and Vietnam all have policies in place to protect the poultry industry by restructuring small producers into production zones or clusters in which improved standards of husbandry and farm biosecurity were to be applied. The APEIR study was designed to measure the impact of this restructuring on the risk of infection and spread of disease, including to humans. The project will be completed in 2013. The findings will improve the organization and management of poultry production zones and contribute to the ongoing policy discussion of the issue.

## Capacity Building

With respect to the second of APEIR's three strategic directions, capacity building, the partnership has seized on a number of opportunities to increase the research capacity of participating research institutions as well as of individual researchers. Meetings and exchanges have enabled the national research teams to jointly design, plan, and implement their projects; and to learn from each other and share their knowledge, skills, and experiences. APEIR researchers have learned how to develop high-quality research proposals and how to harmonize their research so that they can conduct comparative studies.

## Social and Policy Advocacy

Social and policy advocacy work has included producing policy briefs and other publications; holding workshops to present research reports to local authorities; and meeting or consulting with policy makers. As a result of these efforts, APEIR was recognized for its role in fostering regional collaboration at the Association of Southeast Asian Nations (ASEAN)+3 Health Ministers Meeting on Influenza A (H1N1), Bangkok, Thailand (ASEAN 2009). In 2010, APEIR held a media briefing in Kunming, China, that led to news reports around the world. Still, the network could do more. For example, it needs to take greater advantage of its SC members who are senior policy makers and who serve on high-level national committees on EIDs, as they can be effective agents for using relevant research findings to inform and influence policy.

## Case Studies

We have chosen two case studies stories to illustrate the role of the APEIR research partnership in regional emerging infectious disease surveillance across Asia; and how APEIR research activities change course over time. Text Box 1 describes how an APEIR research team assessed links between avian influenza outbreaks in poultry and migratory birds and implications for EID surveillance policy. Text Box 2 illustrates how APEIR policy research has evolved from policy analysis (i.e., factors that influence national pandemic preparedness policy) to policy impact assessment (i.e., the impacts of implemented pandemic policies).

## Relationship to CORDS

APEIR is an active member of Connecting Organizations for Regional Disease Surveillance (CORDS) (11, 12) and collaborates with other regional networks via CORDS in four areas: i) co-organizing with the Mekong Basin Disease Surveillance (MBDS) and other networks to share successful case studies and experience in regional partnership development, including fundraising experiences and policy advocacy; ii) co-funding workshops with other regional networks to disseminate research findings and experiences; iii) interacting with regional diseases surveillance networks by sharing experiences in designing and implementing multi-country, multi-disciplinary and multi-sectoral research projects; and iv) facilitating development of regional-specific research that responds to regional needs in the context of One Health.

## Key Challenges and Lessons Learned

Over its five years, the APEIR partnership has faced several challenges and learned several lessons. Here we elaborate on two key sets of challenges and lessons learned. First, implementing cross-country, multi-institutional research projects takes time because of the harmonization required with respect to both methodology and timing. Harmonization in turn requires timely communication among the national teams, as well as strong leadership and coordination among project team leaders. Despite these challenges, working in partnership provides opportunities that would otherwise not be possible. Additionally, participating researchers are gaining new skills and experience that they can apply to other activities; and both the research institutions involved and the regional network itself are increasing their capacity to conduct similar regional studies in the future.

Second, while combining research with policy is extremely important for establishing a strong regional voice in international debates surrounding EID control debates, influencing policy makers, especially at high levels, can be challenging. APEIR is exerting influence in different ways. For some countries, such as Thailand, at least one of the SC members is in a very influential position to advocate for research-based policy change. Other countries are applying different approaches, such as engaging middle-level policy makers as chairs of steering committee for their projects, involving local policy makers in research, and organizing feedback meetings with local stakeholders.

## Moving Forward

APEIR faces several major challenges to moving forward. Key among these is sustainability. At the time of the formation of APEIR, there were only a few other related networks in the region. Today, five years later, additional networks are emerging and competing for funding. The APEIR partnership is still young and still relies on continuous support from donors. During the first years of its existence, the overwhelming majority of funds for APEIR operations came from IDRC and were determined annually. The Health Systems Research Institute (HSRI), Nonthaburi, Thailand, has also provided significant in-kind contributions in terms of office space for the CO; coordination and communication support; and efforts to organize and convene the SC, regional workshops, and APEIR network meetings. In addition to funds, IDRC also provided key consultative services to help APEIR generate its own resources. As part of its Strategic Plan, 2010–2013, APEIR is striving to diversify its funding base by competing for EID research grants; by seeking more contributions from member countries and institutions; and by seeking funding from other development partners with the mandate and resources for supporting EID research and capacity-building activities in the region.

Another major challenge is that cross-country and Ecohealth/One Health approaches are still quite new to many APEIR members, making it difficult to coordinate agendas and methodologies. Differences in background, culture, and capacity can affect implementation. Related challenges are difficulty in mobilizing the partnership as a whole to generate new research ideas and to prepare high-quality research proposals for funding; and keeping old members and recruiting new members (both individuals and institutions) to the partnership.

Despite these challenges, APEIR has been successful in its early years, demonstrating value in many ways. In terms of APEIR's niche and future role vis-à-vis EIDs in Asia, APEIR's most important value-adding qualities are its multi-country, multi-disciplinary, and multi-sectoral approach; its professionally based but institutionally linked membership; and its strong research-policy interface and emphasis on policy research.
